# Efficacy and factors of myofascial release therapy combined with electrical and magnetic stimulation in the treatment of chronic pelvic pain syndrome

**DOI:** 10.1515/med-2024-0936

**Published:** 2024-05-28

**Authors:** Mingyue Zhu, Fei Huang, Jingyun Xu, Qing Zhou, Bo Ding, Yang Shen

**Affiliations:** Department of Obstetrics and Gynecology, Zhongda Hospital, School of Medicine, Southeast University, Nanjing 210009, Jiangsu, China; Department of Rehabilitation Medicine, Zhongda Hospital, School of Medicine, Southeast University, Nanjing 210009, Jiangsu, China

**Keywords:** myofascial release, trigger points, surface electromyography, dyspareunia

## Abstract

The objective of this study was to evaluate the efficacy and factors of myofascial release therapy combined with electrical and magnetic stimulation in the treatment of chronic pelvic pain syndrome (CPPS). A total of 79 female patients diagnosed with CPPS from January 2021 to December 2022 were prospectively analyzed. Every patient received 3 weeks of treatment which included myofascial release therapy combined with electrical and magnetic stimulation. The visual analog score (VAS) of pelvic floor muscle (PFM) trigger points (TrPs) and the changes in pelvic floor surface electromyography before and after treatment were compared. Multiple linear regression was used to analyze the influencing factors of each outcome index. There were significant differences in VASs of muscle TrPs before and after treatment (*P* < 0.05). For the surface electromyography of PFMs, the differences in pre-baseline rest, post-baseline rest, isometric contractions for muscle endurance evaluation, and coefficient of variation were statistically significant (*P* < 0.05). Linear regression analysis showed that disease course (*X*_1_), dyspareunia (*X*_5_), and urinary incontinence (*X*_6_) were influencing factors for the decline of pre-baseline rest (*r*5 = 1.067, *R* ^2^ = 0.089), post-baseline rest (*r*1 = 0.055, *r*5 = 0.99, *R* ^2^ = 0.119), VASs of ischial spine (*r*5 = 0.916, *R* ^2^ = 0.102), obturator internus (*r*5 = 0.796, *r*6 = −0.703, *R* ^2^ = 0.245), and pubococcygeus (*r*5 = 0.885, *R* ^2^ = 0.149) after treatment in the CPPS group. This study confirmed that individualized myofascial release therapy combined with electrical and magnetic stimulation has significant efficacy for patients with CPPS. At the same time, it is more effective for CPPS patients with longer course of disease, dyspareunia, and without urinary incontinence.

## 1 Introduction

Chronic pelvic pain syndrome (CPPS) is defined as a multifactorial pain disorder that localizes to the anatomic pelvis, anterior abdominal wall at or below the umbilicus, the lumbosacral back, or the buttocks [1,2]. It seriously affects the quality of life and physical and mental health of patients. Structural and functional muscular abnormalities have been suggested as key features of CPPS pathogenesis, specifically hypertonicity of the pelvic floor muscles (PFMs) [3], trigger points (TrPs) in the vulvar area [4], and shortening of the levator ani muscle [5].

TrPs are contracted nodules in a tight band of muscle tissue, a few millimeters in diameter, and can be found in multiple sites in the muscles and myofascial area [6]. The levator ani muscle is one of the most commonly involved PFMs, and 74% of patients with chronic pelvic pain are caused by TrPs [7]. The TrPs can be divided into active TrPs and potential TrPs. The active TrPs can cause pain both in the muscle activity and resting states, and the pain is more persistent and obvious. Potential TrPs usually cause no symptoms or only mild pain. Active TrPs can be converted into potential TrPs when muscles are effectively stretched during daily activities [8,9].

At present, many guidelines for the diagnosis and treatment of pelvic floor dysfunction diseases regard pelvic floor rehabilitation as the first choice of non-surgical treatment [10]. Myofascial release techniques and intravaginal massage can be useful in improving vascularization and releasing muscle TrPs in the pelvic floor and, thus, can be efficient in treating pain and sexual dysfunction [7]. The purpose of electrical stimulation of pelvic pain and PFM overactivity is to reduce pain, relax the overactive PFM, and eliminate symptoms [11]. As a new treatment method, magnetic stimulation can also relax overactive muscles and improve myofascial tension [12]. In addition, magnetic stimulation can reach deep tissues and the stimulation range is deeper and wider [13].

Although CPPS is more common in clinical practice, it is easily overlooked by doctors and patients because of its diverse clinical symptoms, private site of onset, and lack of standard protocols for diagnosis and treatment. At present, there are few studies on the efficacy evaluation of manipulation combined with electrical and magnetic stimulation in the treatment of CPPS, and the efficacy is uncertain. In this study, myofascial release therapy combined with electrical and magnetic stimulation was used to treat CPPS and evaluate the effect of PFM neurophysiology to provide a basis for standardized diagnosis and treatment of CPPS.

## 2 Materials and methods

A total of 87 women with CPPS were recruited from the Center for Women’s Pelvic Floor Health Management at Zhongda Hospital, affiliated to the Southeast University between January 2021 and December 2022.

Inclusion criteria were as follows: (1) age between 18 and 75 years; (2) presence of persistent chronic pelvic pain with visual analog score (VAS) ≥3 points; and (3) during pelvic examination; there was at least one active TrPs in one of the muscle groups, including the obturator internus, levator ani, piriformis, and coccygeus. Exclusion criteria were as follows: (1) the presence of implanted metal or electronic devices close to the site of stimulation; (2) pregnancy status, acute attacks of pelvic inflammatory disease; (3) tumors or other organic lesions of the pelvic organ; (4) psychiatric disorders; and (5) who are undergoing other treatment of chronic pelvic pain.

### 2.1 Measures

All patients were treated with personalized myofascial release therapy to release the tenderness points, and magnetic stimulation/electrical stimulation was performed alternately after 5 min of rest. The efficacy was evaluated by comparing the pelvic floor surface electromyography and the VAS of PFM according to the pain maps before and after treatment. Outcome assessments were performed by a professional physician, interventions were performed by a physical therapist with professional training, and all data analyses were performed by another physician. We ensured that blinding measures were put in place for the physicians involved in conducting outcome assessments and analyzing the data. All interventions were conducted during non-menstrual periods.

#### 2.1.1 Myofascial release therapy

PFM relaxation training was performed first: as the index finger entered the vagina, the pulse in the muscle felt by the finger suggested that the PFMs were fully relaxed. Then, the TrPs of the muscle were found by the painful map score for massage and relaxation treatment: the finger continued to press the tender point for 8–10 s (can be repeated many times) and the pressure could be gradually increased as the pain was reduced. When the tension of the TrPs decreased or was no longer sensitive, the pressure could be removed.


**Informed consent:** Participants who were willing to participate and had signed informed consent were assessed for eligibility.
**Ethical approval:** The protocol was approved by the Clinical Research Ethics Committee of Zhongda Hospital (protocol registration no. 2022ZDSYLL107-P01). This study was performed following the CONSORT guideline and in accordance with the Declaration of Helsinki.

#### 2.1.2 Intravaginal electrical stimulation

The pelvis and abdomen were fully exposed, and the electrodes were placed in the vagina and hypogastrium to form an electrical circulation using the Vishee neuro-muscle stimulator (MyoTrac Infiniti, model SA9800, Thought Technology Ltd). Electrical stimulation was given for 30 min each. The frequency was adjusted to 50 Hz, the duration was 2 s, the wave width was 250 μs, and the current intensity was increased from 0 which was appropriate for the patient to feel muscle contraction without discomfort and maintain the current intensity stimulation (less than 50 mA).

#### 2.1.3 Magnetic stimulation

The patient sat directly in the center of the coil of the magnetotherapy chair (the Vishee instrument, model Magneuro60), and the stimulation intensity was adjusted according to the patient’s subjective feeling. During every treatment, the first intensity needed to make the patient’s groin has a sense of contraction, and the next appropriate intensity is the intensity that produces a sense of contraction plus 5–10% (the stimulation frequency was 10 Hz, the stimulation time was 4 s, the rest time was 6 s, and the treatment time was 20 min).

All the patients were treated at intervals every 2 days for 3 weeks (myofascial release therapy was performed 10 times while electrical stimulation and magnetic stimulation were performed 5 times respectively in total).

### 2.2 Outcome measures

Participants' pain map and surface electromyography of PFMs were evaluated at two time points: (1) baseline before intervention and 3 weeks post-intervention.

#### 2.2.1 PFM pain map

To assess the palpation points of CPPS, each point was manipulated and examined [14,15]. These specific points were located deep within the PFM groups and included the bilateral coccygeus, iliococcygeus, ischial spine, pubococcygeus, puborectalis, piriformis, and obturator internus, as well as one point at the anococcygeal raphe. The palpation pressure of 0.4–0.5 kg/cm^2^ was applied. During the assessment of each muscle, the patient was asked to give VAS while she reported pain.

#### 2.2.2 Surface electromyography of PFMs

The Glazer Protocol was used to record the bioelectrical activity of the PFM by intravaginal electrodes, which included a series of contractions and relaxations of muscle described below [16]: pre-baseline rest, phasic contractions, tonic contractions, isometric contractions for muscle endurance evaluation, and post-baseline rest.

### 2.3 Statistical analyses

For data analysis, SPSS 26.0 was used. Categorical variables were described by the number of cases (percentage). Continuous variables were described as median (interquartile range)/mean ± SD. Measurement data (not in accordance with normal distribution) were analyzed by Wilcoxon rank-sum test. Standard multiple linear regression was used to analyze the influence of demographic variables on the efficacy analysis of women with CPPS. *P* < 0.05 was considered statistically significant.

## 3 Results

### 3.1 Participant characteristics

A total of 8 patients were excluded because of various reasons, including not meeting the inclusion criteria, declining to participate, and other reasons. Seventy-nine patients met the inclusion criteria in total and underwent the intervention without any participant withdrawing or dropping off from the study. The median age was 30 years, ranging from 24 to 72 years. Patients reported that they had been diagnosed with CPPS 9 (7,12) months before the study began (range, 6–54 months).

### 3.2 Changes in the PFMs

Each patient had multiple TrPs of the PFMs before treatment and the VASs of all PFMs were ≥3. The *P* value of PFMs’ TrPs was less than 0.05 after the treatment (Table 1), and the difference was statistically significant, suggesting that the degree of patients’ PFMs pain was greatly relieved after intervention.

**Table 1:** Changes in the VASs of pelvic floor muscle

Variable	Before	After	*P*-value
AR	3 (3.3)	1 (0.1)	<0.001
Pir	5 (4.6)	2 (1.3)	<0.001
C	5 (4.7)	1 (0.3)	<0.001
IS	6 (4.7)	2 (1.3)	<0.001
IC	5 (4.6)	1 (1.2)	<0.001
PR	5 (4.6)	1 (0.2)	<0.001
OI	5 (4.6)	1 (0.2)	<0.001
PC	3 (2.4)	1 (0.2)	<0.001

AR: anococcygeal raphe, Pir: piriformis, C: coccygeus, IS: ischial spine, IC: iliococcygeal muscle, PR: puborectalis, OI: obturator internus, PC: pubococcygeus.

For the surface electromyography of PFMs, the differences in pre-baseline rest, post-baseline rest, isometric contractions for muscle endurance evaluation, and coefficient of variation (CV) were statistically significant (*P* < 0.05), while there was no significant difference in the phasic contractions and tonic contractions (*P* > 0.05) after the treatment, indicating that the tension, endurance and stability of PFMs in all the patients with intervention were significantly improved (Table 2).

**Table 2:** Changes in the surface electromyography of pelvic floor

Variable		Before	After	*P*-value
Pre-baseline rest	Mean (μV)	6.10 (4.89,7.87)	4.07 (3.54,5.21)	<0.001
	CV	0.12 (0.06,0.18)	0.08 (0.06,0.12)	0.003
Phasic contractions	Maximum (μV)	32.37 (23.23,45.26)	34.00 (30.24,36.76)	0.634
	Relax time	0.98 (0.72,1.72)	0.45 (0.34,0.85)	<0.001
Tonic contractions	Mean (μV)	25.48 (19.43,39.70)	30.11 (25.67,32.67)	0.239
	CV	0.26 (0.23,0.32)	0.21 (0.16,0.26)	<0.001
	Relax time	1.19 (0.84,2.19)	0.67 (0.46,1.01)	<0.001
Isometric contractions for muscle endurance evaluation	Mean (μV)	23.30 (17.45,28.77)	28.09 (23.56,30.59)	0.001
	CV	0.21 (0.16,0.27)	0.16 (0.13,0.19)	<0.001
Post-baseline rest	Mean (μV)	6.15 (5.20,8.48)	3.97 (3.26,5.03)	<0.001
	CV	0.12 (0.07,0.19)	0.10 (0.06,0.13)	0.001

CV: coefficient of variations.

Through the urine leakage test, we found that this treatment can improve urinary leakage condition (Table 3).

**Table 3:** Changes in the 1 h urine pad test urine leakage (g)

Variable	Before	After	*P*-value
1 h urine pad test urine leakage	6.56(5.32,7.38)	2.63 (2.21,4.05)	<0.001

A 1 h urine pad test: a standardized quantitative method for urine loss which was used to assess the symptoms of urinary incontinence through the amount of urine lost. Participants put on a preweighed pad, drank 500 mL water in 15 min, and then performed a series of activities. Afterward, the pad was weighed again to measure the amount of urine leakage.

### 3.3 Analysis of factors affecting the efficacy of the intervention

Based on the literature review and experience, the factors that may affect the physical therapy of CPPS are as follows: the disease course (*X*_1_), age (*X*_2_), BMI (*X*_3_), dysmenorrhea (*X*_4_), dyspareunia (*X*_5_), and urinary incontinence (UI) (*X*_6_) were used as independent variables, and the difference of each outcome index Δ*y* (the outcome index value before treatment was *y*1, the outcome index value after treatment was *y*2, Δ*y* = *y*2 − *y*1 was used as the dependent variable for multiple linear regression analysis. The linear regression model between the differences of each variable and each efficacy index was finally obtained (Table 4):

**Table 4:** Results of variable selection in the intervention group

Δ*Y*	Δ*X*	*B*	Std. error	*t*	*P*-value	95% CI	*R*²	*F*
Δpre-baseline rest	cons	1.894	0.253	7.49	0	1.39–2.397	0.089	*F* = 8.574, *P* = 0.004
	*X*_5_	1.067	0.365	2.928	0.004	0.342–1.793		
Δpost-baseline rest	cons	1.709	0.319	5.362	0	1.074–2.343	0.119	*F* = 6.286, *P* = 0.003
	*X*_1_	0.055	0.021	2.556	0.013	0.012–0.097		
	*X*_5_	0.99	0.402	2.459	0.016	0.188–1.791		
ΔIS	cons	3.268	0.203	16.121	0	2.865–3.672	0.102	*F* = 9.818, *P* = 0.002
	*X*_5_	0.916	0.292	3.133	0.002	0.334–1.498		
ΔOI	cons	3.323	0.182	18.212	0	2.96–3.684	0.245	*F* = 13.642, *P*＜0.001
	*X*_5_	0.796	0.229	3.474	0.001	0.339–1.252		
	*X*_6_	−0.703	0.259	−2.714	0.008	−1.219–(−0.187)		
ΔPC	cons	1.878	0.16	11.706	0	1.559–2.198	0.149	*F* = 14.640, *P*＜0.001
	*X*_5_	0.885	0.231	3.826	<0.001	0.424–1.346		

UI: urinary incontinence, IS: ischial spine, OI: obturator internus, PC: pubococcygeus, CI: confidence interval, *B*: beta coefficient.

ΔPre-baseline rest = 1.894 + 1.067*X*_5_ (*F* = 8.574, *P* = 0.004, *N* = 79, *R*² = 0.089),

ΔPost-baseline rest = 1.709 + 0.055*X*_1_ + 0.99*X*_5_ (*F* = 6.286, *P* = 0.003, *N* = 79, *R*² = 0.119),

ΔIS = 3.268 + 0.916*X*_5_ (*F* = 9.818, *P* = 0.002, *N* = 79, *R*² = 0.102),

ΔOI = 3.323 + 0.796*X*_5_ − 0.703*X*_6_ (*F* = 13.642, *P*＜0.001, *N* = 79, *R*² = 0.245),

ΔPC = 1.878 + 0.885*X*_5_ (*F* = 14.640, *P*＜0.001, *N* = 79, *R*² = 0.149).

For pelvic floor electromyography, the adjusted coefficient of determination R showed that dyspareunia could explain 8.90% of the total variation of the decline degree of pre-baseline rest in CPPS patients after treatment, and the correlation coefficient *r* > 0, indicating that patients with dyspareunia had relatively good curative effect. Disease course and dyspareunia could explain 11.90% of the total variation of decline of post-baseline rest in patients with CPPS after treatment, and patients with longer course of disease and dyspareunia had relatively better efficacy. Similarly, for the TrPs of PFMs, the correlation coefficient showed that patients with dyspareunia and without urinary incontinence had relatively better efficacy.

## 4 Discussion

Our study confirmed that individualized myofascial release therapy combined with electrical and magnetic stimulation for the treatment of CPPS significantly reduced the intensity of PFM pain in patients, while improving neuromuscular activity and stability of PFMs and reducing the hypertonia of muscles. Myofascial release therapy can make local vessels expand, accelerate blood flow and lymphatic return, reduce inflammatory exudation, reduce edema symptoms, and quickly absorb inflammatory products, so as to relieve pelvic pain [17,18].

From the results of pelvic floor electromyography before and after treatment in our study, pre- and post-baseline rest values were significantly decreased, suggesting that the tension of overactive PFMs decreased. The changes in isometric contractions for muscle endurance evaluation suggested that the endurance of PFMs was improved, and the changes in CV also indicated that the treatment improved the stability of the PFMs. Electrical stimulation reduces the sensitivity of PMFs by inhibiting parasympathetic arousal of the pelvic floor [19]. It can also reduce the concentration of metabolites in tissue fluid around muscle fibers, increase the proportion of anti-fatigue muscle fibers in the PFMs, and improve muscle endurance [20]. The electromagnetic field goes non-invasively through the neuromuscular tissue, where the induced electric currents depolarize the neural cells, thus altering the resting membrane potential and, thereby, reducing the transmission of painful impulses [21,22]. Magnetic stimulation of the pelvis produces a direct stimulus in muscular trophism, favoring an anti-inflammatory effect, in addition to its relaxing and de-contracting effect, as it reduces the sympathetic tone and restores the normal muscular activity of the pelvic floor [23]. Kim et al. [24] have investigated the effect of extracorporeal magnetic stimulation on symptoms of CPPS and offered a new treatment option for patients with CPPS who do not respond to pharmacotherapy. Wu et al. [25] found that biofeedback and electrical stimulation combined with prostate massage has a synergistic effect on CP/CPPS by alleviating pain and urinary symptoms and improving the quality of life. The combination of magnetic and electrical stimulation can effectively enhance muscle strength, improve pelvic floor function, promote local tissue blood circulation, and regulate damaged pelvic floor nerves. All of the above indicate the necessity and importance of electrical and magnetic stimulation in the treatment of CPPS.

This study is the first to find that the disease course, dyspareunia, and urinary incontinence were associated with the efficacy of myofascial release therapy combined with electrical and magnetic stimulation in the treatment of CPPS using multiple linear regression models. The longer the course of CPPS, the contracture of muscle fiber makes the PFMs in a state of high tension and hypoxia for a long time, which can lead to the release of sensitive substances in the TrP area [26], resulting in the increased sensitivity of local pain receptors [27] and obvious TrP pain. Meanwhile, repeated or prolonged somatic and visceral sensory input of nociceptors, resulting in lowering their activation threshold, and sensitization of previously non-involved afferent nerve fibers [28,29]. In addition to overactivity, dyspareunia is related to a decrease in the strength and endurance of PFMs [30]. Therefore, myofascial release therapy combined with electrical and magnetic stimulation for different tenderness points is more effective for CPPS patients with longer course of disease and dyspareunia. A variety of pathogenic factors can cause pathological changes such as imbalance of extracellular matrix metabolism, oxidative stress and hypoxia in the nerve, muscle and connective tissue of pelvic floor, leading to the urinary continence nerve injury or weak pelvic structure support and function, which leads to urinary incontinence [31,32]. For CPPS patients with urinary incontinence, the treatment in this study further reduced the tense and activity of PFM tone to some extent, which affected the efficacy to some degree.

### 4.1 Strengths and limitations

Compared with other studies, our study provided a comprehensive evaluation and treatment for CPPS patients through the release of local TrPs of PFMs and the stimulation of superficial and deep PFMs and nerves. The use of muscle TrP VAS combined with surface electromyography data of PFM tension, contraction, endurance, and stability as efficacy evaluation can not only clarify the different muscle sources of pain points but also quantify the outcome indicators, making the data more objective and reliable. Our study is also the first to explore the factors associated with the effectiveness of this combined intervention to identify populations more suitable for this treatment, providing a new, potentially valid, therapeutic alternative for the management of patients with CPPS.

Inevitably, our study exhibits the following limitations. There are several limitations to the current study. First, because the patients were from the same medical unit, there would be some bias in selection. Second, the study lacked a placebo group. In addition, this study aimed to evaluate the efficacy in a short period of time, and a larger sample size and longer follow-up may be needed to further evaluate the efficacy results in the future.

## 5 Conclusion

This study confirmed that individualized myofascial release therapy combined with electrical and magnetic stimulation has significant efficacy for patients with CPPS. At the same time, it is more effective for CPPS patients with longer course of disease, dyspareunia, and without urinary incontinence.

## Abbreviations

CPPS: chronic pelvic pain syndrome
AR: anococcygeal raphe
Pir: piriformis
C: coccygeus
IS: ischial spine
IC: iliococcygeal muscle
PR: puborectalis
OI: obturator internus
PC: pubococcygeus
